# A Pediatric Case of Cowden Syndrome with Graves' Disease

**DOI:** 10.1155/2017/2750523

**Published:** 2017-01-31

**Authors:** Cláudia Patraquim, Vera Fernandes, Sofia Martins, Ana Antunes, Olinda Marques, José Luís Carvalho, Jorge Correia-Pinto, Carla Meireles, Ana Margarida Ferreira

**Affiliations:** ^1^Pediatrics Department, Hospital de Braga, Sete Fontes, São Victor, 4710-243 Braga, Portugal; ^2^Endocrinology Department, Hospital de Braga, Sete Fontes, São Victor, 4710-243 Braga, Portugal; ^3^Life and Health Sciences Research Institute (ICVS), School of Health Sciences, University of Minho, Braga, Portugal; ^4^ICVS/3B's-PT Government Associate Laboratory, Braga, Guimarães, Portugal; ^5^Pediatric Endocrinology Department, Hospital de Braga, Sete Fontes, São Victor, 4710-243 Braga, Portugal; ^6^Pediatric Surgical Department, Hospital de Braga, Sete Fontes, São Victor, 4710-243 Braga, Portugal; ^7^Pediatrics Department, Hospital da Senhora da Oliveira, Creixomil, 4835-044 Guimarães, Portugal; ^8^Anatomic Pathology Department, Hospital de Braga, Sete Fontes, São Victor, 4710-243 Braga, Portugal

## Abstract

Cowden syndrome (CS) is a rare dominantly inherited multisystem disorder, characterized by an extraordinary malignant potential. In 80% of cases, the human tumor suppressor gene phosphatase and tensin homolog (PTEN) is mutated. We present a case of a 17-year-old boy with genetically confirmed CS and Graves' disease (GD). At the age of 15, he presented with intention tremor, palpitations, and marked anxiety. On examination, he had macrocephaly, coarse facies, slight prognathism, facial trichilemmomas, abdominal keratoses, leg hemangioma, and a diffusely enlarged thyroid gland. He started antithyroid drug (ATD) therapy with methimazole and, after a 2-year treatment period without achieving a remission status, a total thyroidectomy was performed. Diagnosis and management of CS should be multidisciplinary. Thyroid disease is frequent, but its management has yet to be fully defined. The authors present a case report of a pediatric patient with CS and GD and discuss treatment options.

## 1. Introduction

Cowden syndrome (CS) is a rare dominantly inherited multisystem complex disorder with high variability and susceptibility, incomplete penetrance, and an identifiable germline mutation [1–4]. The human tumor suppressor gene, phosphatase and tensin homolog (PTEN), is mutated in 80% cases; mutations in other genes such as KILLIN, SDH B/D, PIK3CA, and AKT1 are responsible for the rest of the cases [4]. After gene identification, the incidence of CS was calculated to be 1 in 200,000 [2, 5, 6], which is considered underestimated given the common manifestations and variable expression of the syndrome [5].

CS was first described in 1963 by Lloyd and Dennis referring to their 20-year-old patient who died of breast cancer, Rachel Cowden, after whom the syndrome was named [2–6]. A slight female predominance appears to be present [2, 7]. It usually occurs in the second or third decade of life and generally involves the skin, oral mucosa, thyroid, breast, gastrointestinal tract, genitourinary tract, central nervous system, and bone, being characterized by an extraordinary potential for malignant transformation [4–7].

Mucocutaneous manifestations, apparent in 99-100% of patients, are a pathognomonic feature and usually the first presenting lesions of the disease. These lesions include trichilemmomas (hamartomas of the infundibulum of the hair follicle), facial papules, acral keratoses, oral cavity verrucous or papillomatous papules, benign (e.g., angiomas, dermal fibromas, lipomas, and neurinomas) and malignant tumors (e.g., melanomas, basal cell carcinoma, and squamous cell carcinomas) [4–7]. Extracutaneous manifestations occur in about 90% of cases, most often involving the thyroid gland (50–70%), with the majority of lesions being adenomatous goiters or multiple follicular adenomas [4, 5]. Thyroid carcinoma has been reported in 3–10%, mostly follicular or papillary carcinomas, but medullary carcinoma may also occur, even in children. Other frequent lesions related to CS are hamartomatous polyps of the digestive tract, fibrocystic disease of the breast, uterine leiomyoma, and macrocephaly [5, 8].

Graves' disease (GD) is an autoimmune disease that affects the thyroid gland and includes diffuse goiter, hyperthyroidism, and ophthalmopathy. Pathophysiology of GD involves a complex interplay between genetic and environmental influences, but the exact mechanisms are not yet known. GD is a rare condition in childhood, with an estimated incidence of 0,1–3 per 100,000. Antithyroid drugs (ATDs) are generally considered the first-line treatment. Nevertheless, long-term remission rates after 2 or more years of ATDs have been reported to be low and definitive treatment modalities with radioiodine (RAI) ablation or total thyroidectomy may be necessary [9].

There are no evidence-based guidelines for management of these conditions in childhood. Although patients with CS are known to have a predisposition for thyroid disease, the exact incidence and pathophysiology are still undefined, as is the best way to manage those children and adolescents [5].

To the best of our knowledge, reports of pediatric patients with CS and GD are exceedingly rare in literature. The authors present a case report of a pediatric patient with CS and GD and discuss treatment options.

## 2. Case Description

The patient is a 17-year-old Caucasian boy that presented with intention tremor, palpitations, and marked anxiety at age 15. He was medicated with propranolol and hydroxyzine with partial improvement of symptoms and was referred to a hospital pediatric appointment.

The adolescent and his father had genetically confirmed CS [heterozygous frameshift mutation in exon 5 of PTEN gene: c.405_406insA (C136Mfs*∗*44) NM_000314]. The patient's father was submitted to a total thyroidectomy for multinodular goiter at age 23 and died at 60 years old of brain tumor (anaplastic oligodendroglioma). His paternal grandfather had thyroid cancer.

**Figure 1:**
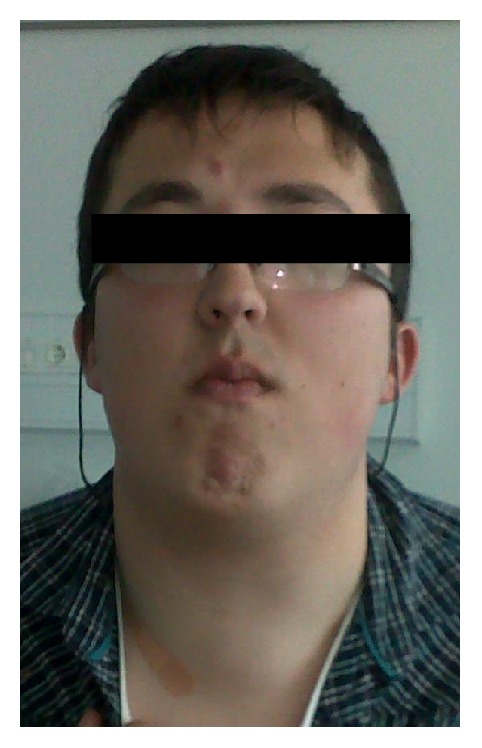
Patient photograph showing diffusely enlarged thyroid gland (frontal view).

**Figure 2:**
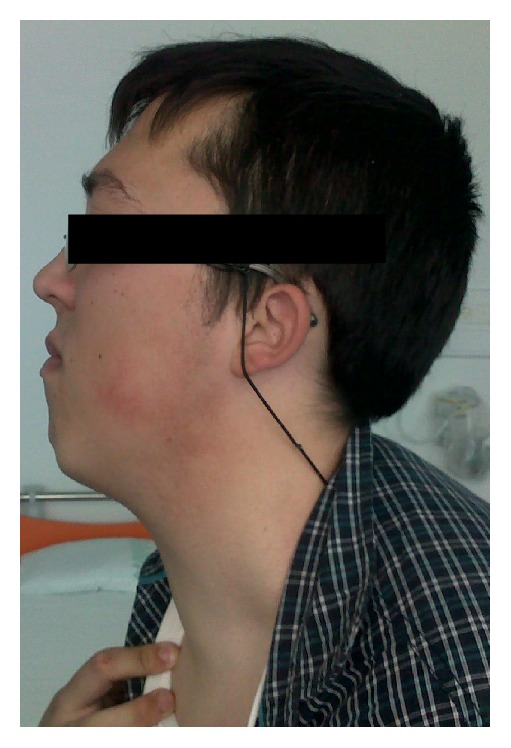
Patient photograph showing diffusely enlarged thyroid gland (left profile).

Other notable features in our patient's previous medical history were macrocephaly, the presence of a hemangioma in his left lower limb, learning disabilities, and a cognitive evaluation that concluded that a global delay in cognitive development with an intelligence quotient (IQ) at the lower limit of the normal range was present. On physical examination, he had a coarse facies, slight prognathism, facial trichilemmomas, abdominal keratoses, and an irregular, elastic, and diffusely enlarged thyroid gland (Figures 1 and 2). Exophthalmia was not apparent.

Thyroid function tests were consistent with a hyperfunctioning thyroid state: thyrotropin (TSH) < 0,005 IU/mL (normal range 0,358–3,740 IU/mL), free thyroxine (fT4) 4,97 ng/dL (normal range 0,76–1,46 ng/dL), free triiodothyronine (fT3) 27,58 pg/mL (normal range 2,18–3,98 pg/mL), with positive anti-thyroid autoantibodies: thyroid peroxidase antibodies (anti-TPO) > 3340 IU/mL (normal range 0–100 IU/mL), thyroglobulin antibodies (anti-Tg) > 24400 IU/mL (normal range 0–344 IU/mL), and thyrotropin receptor antibodies (TRAbs) 14,3 IU/mL (normal range < 1,2 IU/mL). Thyroid ultrasonography at diagnosis showed a heterogeneous, hypoechoic, and high volume goiter (43 × 94 × 38 mm left lobe and 49 × 100 × 44 mm right lobe), with no visible nodules.

He was started on methimazole, maximum dose 20 mg/day, and propranolol treatment, lasting for a 2-year period, without achieving an euthyroid state.

**Figure 3:**
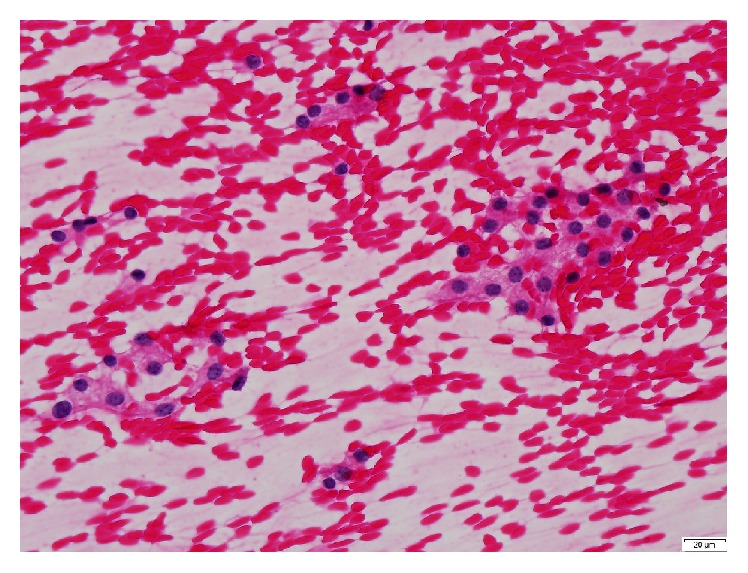
Fine needle aspiration (FNA) cytology: benign, consistent with hyperplastic/adenomatoid nodule (H&E, 400x).

Thyroid ultrasonography, 2 years after GD diagnosis, revealed dominant isoechoic solid nodules of 10 mm and 12 mm in the left and right lobes, respectively. Fine needle aspiration (FNA) cytology result was benign, consistent with hyperplastic/adenomatoid nodule (designed according to The Bethesda System for Reporting Thyroid Cytopathology) (Figure 3).

**Figure 4:**
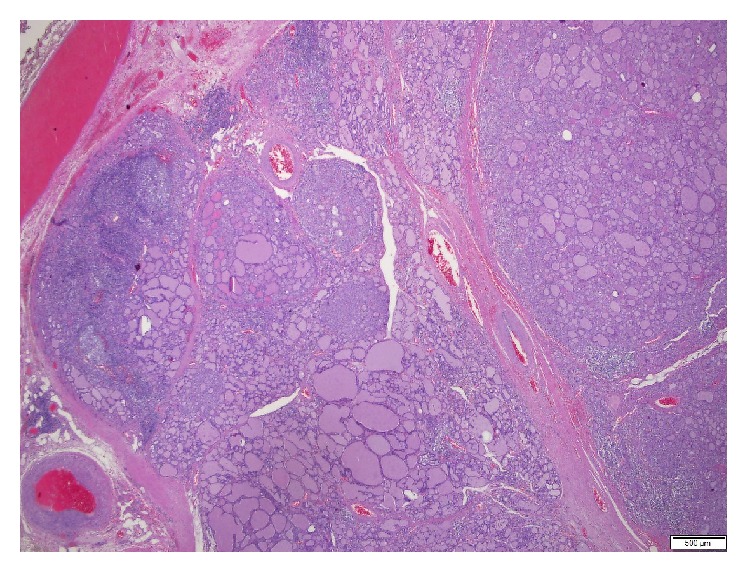
Thyroid gland presenting with lymphocytic thyroiditis and multinodular goiter (H&E, 20x).

**Figure 5:**
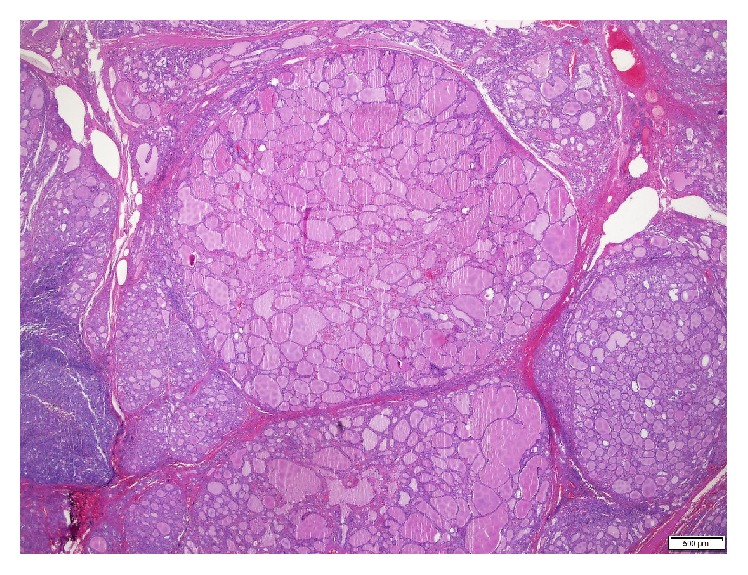
Multinodular goiter (H&E, 20x).

**Figure 6:**
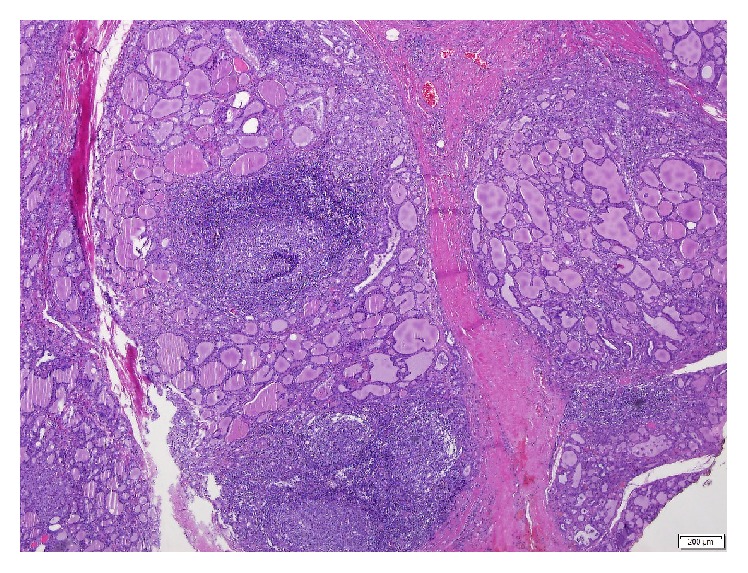
Lymphocytic thyroiditis (H&E, 40x).

A multidisciplinary team, including Pediatric Endocrinologist, Adult Endocrinologist, and Pediatric Surgeon, met in order to discuss the therapeutic options. Attending to the voluminous goiter and risks inherent to CS, a total thyroidectomy was the definite treatment consensus. That was also the patient's choice. Potassium iodide-iodine solution (Lugol) was used in preoperative preparation and surgery went uneventful. On gross examination, the surgical specimen weighed 218 g, with a larger left (95 mm) than right lobe (90 mm). The microscopic examination revealed lymphocytic thyroiditis (Figures 4–6). He was started on replacement therapy with oral levothyroxine 125 ug/day and he is currently compensated.

## 3. Discussion

Early recognition of individuals with CS is essential in order to start appropriate cancer screening and prevent potential complications associated with this disorder [1, 4].

Thyroid disease is an important concern among patients with CS [5]. Clinical guidelines from the National Comprehensive Cancer Network (NCCN) recommend that patients with CS should undergo an annual physical examination starting at the age of 18 years or 5 years before the youngest age of diagnosis of a component cancer in the family (whichever is earlier), with special care to thyroid examination. They also advocate an annual thyroid ultrasound since the age of 18 years or 5–10 years before the youngest age of diagnosis of a thyroid cancer in the family (whichever is younger) [10]. On the other hand, some authors recommend that all patients with CS should undergo baseline thyroid ultrasound at the age of diagnosis, with follow-up on a yearly basis, given that the risk of thyroid cancer begins early in childhood and that ultrasound is an innocuous screening procedure [3]. Other studies advocate that thyroid examinations and ultrasound should be performed from the age of 10 years [6]. Thyroid surgery, if needed, ought to be a total thyroidectomy, even if only one side of the thyroid seems to be affected, owing to the high probability for additional disease and need for future reoperation [1, 3]. Prophylactic thyroidectomy has been considered an option for selected patients in whom lifetime screening may be challenging (e.g., patients with autism or other developmental disorders) [1, 3]. It may also be reasonable for CS patients who have nodules and understand the risks associated with this surgical procedure [1].

For GD, definitive treatment options are RAI or total thyroidectomy. RAI has been increasingly used in children except for the very young (less than 5 years of age), although theoretical concerns of heightened cancer risk still persist. Surgical treatment is most commonly used in the following situations: very large goiters (more than 80 g), children younger than 5 years, planned pregnancy, or patient's choice [9]. Currently, the ideal therapeutic approach remains controversial.

Regarding our patient and the available therapeutic options, RAI could be related to an increased cancer risk, which is of special concern in CS; possible surgical complications include anesthetic risk, recurrent laryngeal nerve injury, hypoparathyroidism, and wound infection [9]. The experience of treatment centers and specific characteristics of each patient are definitely decisive factors in the therapeutic choice.

In the present case, it was decided to perform a total thyroidectomy given the size of the goiter, increased risk of malignancy inherent to CS, and patient's will.

Lugol's solution was used in the preoperative preparation to reduce thyroid gland vascularity and potential surgical blood loss [11].

To the best of our knowledge, reports of pediatric patients with CS and GD are extremely uncommon. A meta-analysis from Hall and coworkers reported only 1 case of hyperthyroidism among 96 patients with CS and thyroid disease [5].

In conclusion, the diagnosis and the management of CS should be multidisciplinary. This syndrome has significant malignant associations, therefore requiring early clinical suspicion and aggressive screening. Thyroid disease occurs in about two-thirds of patients, being one of the most frequent extracutaneous manifestations, thus demanding special attention and careful monitoring. Despite that, the management of thyroid disease in CS has yet to be fully defined. Furthermore, meticulous evaluation of patient's family is essential, not only for identification of potential at risk relatives but also for genetic counseling.

## Abbreviations

anti-Tg: Thyroglobulin antibodies
anti-TPO: Thyroid peroxidase antibodies
ATD: Antithyroid drug
CS: Cowden syndrome
FNA: Fine needle aspiration
fT3: Free triiodothyronine
fT4: Free thyroxine
GD: Graves' disease
IQ: Intelligence quotient
NCCN: National Comprehensive Cancer Network
PTEN: Phosphatase and tensin homolog
RAI: Radioiodine
TRAbs: Thyrotropin receptor antibodies
TSH: Thyrotropin.
